# The PNA mouse may be the best animal model of polycystic ovary syndrome

**DOI:** 10.3389/fendo.2022.950105

**Published:** 2022-08-08

**Authors:** Jingyi Ren, Guangqing Tan, Xinyi Ren, Weiyu Lu, Qiling Peng, Jing Tang, Yingxiong Wang, Biao Xie, Meijiao Wang

**Affiliations:** ^1^ Department of Physiology, College of Basic Medicine, Chongqing Medical University, Chongqing, China; ^2^ College of Basic Medicine, Chongqing Medical University, Chongqing, China; ^3^ Joint International Research Laboratory of Reproduction and Development of the Ministry of Education of China, College of Public Health and Management, Chongqing Medical University, Chongqing, China; ^4^ Department of Bioinformatics, College of Basic Medicine, Chongqing Medical University, Chongqing, China; ^5^ Department of Biostatistics, School of Public Health and Management, Chongqing Medical University, Chongqing, China

**Keywords:** polycystic ovary syndrome (PCOS), bioinformatics, animal model, dehydroepiandrosterone (DHEA), differentially expressed genes (DEG), RNA sequencing (RNA-seq)

## Abstract

Polycystic ovary syndrome (PCOS) exerts negative effects on females of childbearing age. It is important to identify more suitable models for fundamental research on PCOS. We evaluated animal models from a novel perspective with the aim of helping researchers select the best model for PCOS. RNA sequencing was performed to investigate the mRNA expression profiles in the ovarian tissues of mice with dehydroepiandrosterone (DHEA) plus high-fat diet (HFD)-induced PCOS. Meanwhile, 14 datasets were obtained from the Gene Expression Omnibus (GEO), including eight studies on humans, three on rats and three on mice, and genes associated with PCOS were obtained from the PCOSKB website. We compared the consistency of each animal model and human PCOS in terms of DEGs and pathway enrichment analysis results. There were 239 DEGs shared between prenatally androgenized (PNA) mice and PCOS patients. Moreover, 1113 genes associated with PCOS from the PCOSKB website were identified among the DEGs of PNA mice. A total of 134 GO and KEGG pathways were shared between PNA mice and PCOS patients. These findings suggest that the PNA mouse model is the best animal model to simulate PCOS.

## Introduction

Polycystic ovary syndrome (PCOS) is an endocrine disorder characterized by hyperandrogenemia, ovulatory dysfunction and metabolic abnormalities and is common among childbearing-aged females (1–4). PCOS has a negative impact on 5-10% of women (2, 5), but its pathogenesis is still unclear (6). Despite the differences in reproductive physiology between experimental animals and humans (7), the limitations related to ethical issues and specifically to obtaining material for research in humans are undeniable. The ease of obtaining and raising experimental animals has also led to a greater use of animal models in basic research on the pathophysiology of PCOS (8, 9).

Various animal models of PCOS have been explored and studied for over 60 years (8). According to the Rotterdam criteria for PCOS diagnosis (2), the main features of PCOS are excess androgens, increased numbers of cystic follicles, and abnormal menstrual cycles (estrous cycles in animal models). The widely used animal models of PCOS include the androgen model (10, 11), estrogen model (12, 13), aromatase inhibitor model (14, 15) and combined models, such as the dehydroepiandrosterone (DHEA) plus high fat diet (HFD)-induced mouse model (16), high-fat high-sugar (HFHS)-induced mouse model (17, 18), etc. Although a variety of PCOS animal models have been established for research purposes, there is still disagreement which model best recapitulates the disease (19, 20). Thus, the choice of an optimal model remains an important issue.

Whether the reproductive characteristics of the model meet the Rotterdam criteria for PCOS diagnosis and their degree of similarity to the characteristics of PCOS patients are the main concerns for researchers in selecting the best model. However, there is still controversy about which model is best because no single animal model exhibits all of the key pathophysiological features of patients with PCOS. Bioinformatics analysis of data from many different studies of PCOS could help us determine how the potential mechanisms cause phenotypic alterations.

Therefore, it is necessary to evaluate PCOS animal models from a novel perspective. In this study, we compared the eight groups of PCOS animal models’ data (our data the mRNA expression profile of a DHEA plus HFD-induced PCOS mouse model, and seven groups’ data from public databases) with PCOS patients’ data to identify the consistency of differentially expressed genes (DEGs) of PCOS and control, respectively. It provides new insights and reliable references for researchers when selecting models.

## Materials and methods

**Figure 1:**
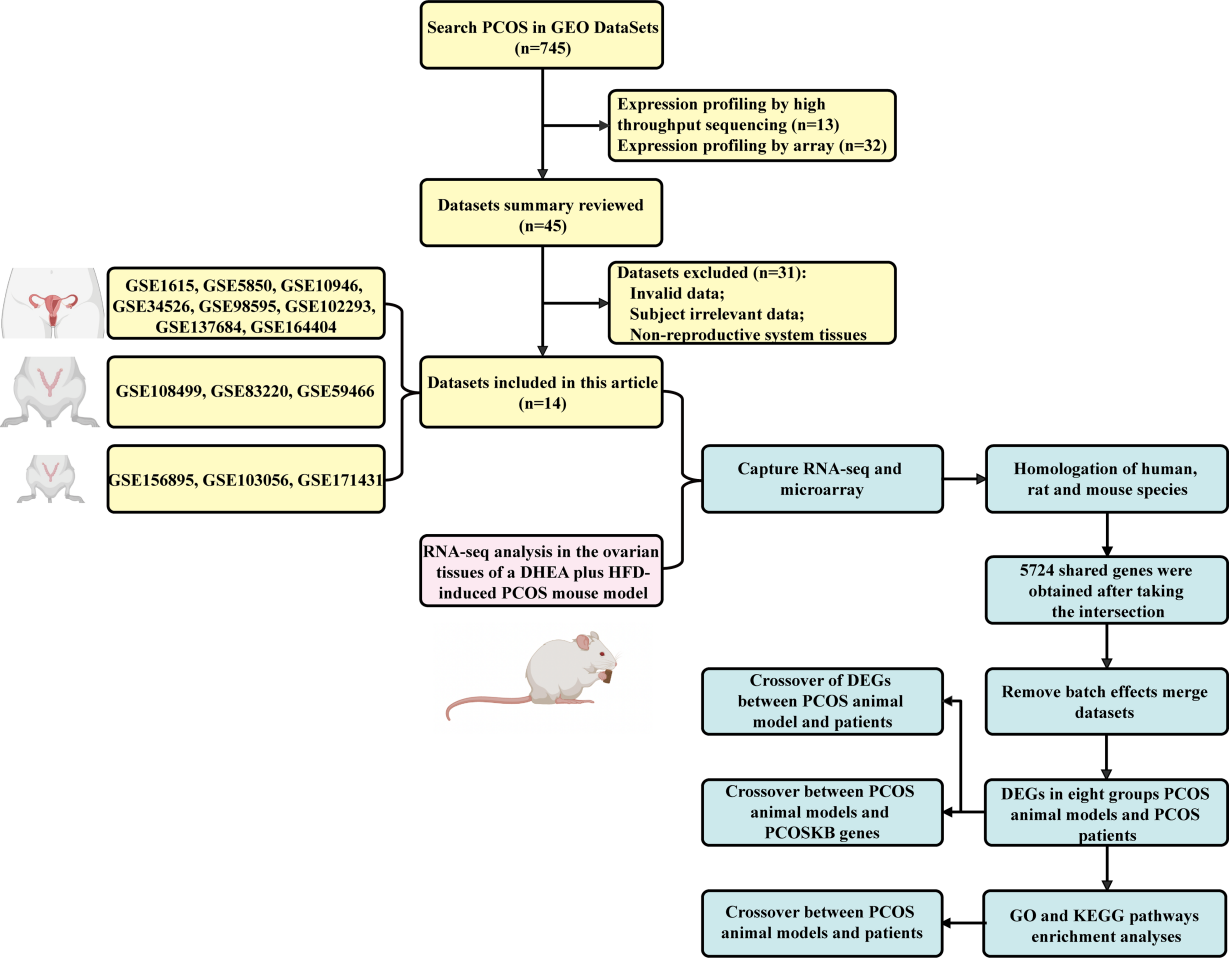
The workflow used in this study is illustrated.

The workflow diagram is illustrated in **Figure 1**.

### Animal experiments

All animal studies were approved by the Ethics Committee of Chongqing Medical University. PCOS model mice were established as described by Wang et al. (21). Twenty-one-day-old female C57BL/6J mice were obtained from the animal research center of Chongqing Medical University. All animals were maintained under standard housing conditions (20 °C and 12 h day/night cycles) with free access to water and food according to the institutional guidelines. After four days of acclimatization, the mice were randomly divided into two groups (a control group of 25 mice and a PCOS group of 20 mice). The mice in the PCOS group were injected with DHEA (6 mg/100 g/d) dissolved in 0.1 ml of sesame oil daily and fed a HFD (60% of calories from fat). The control mice were injected with 0.1 ml of sesame oil daily and fed normal chow.

### Vaginal smears and estrous cycle determination

The estrous cycle was examined by vaginal smears. Inspections were performed daily at 9:00 a.m. for ten consecutive days before sacrifice. The estrous cycle was determined by analysis of vaginal smears under a microscope. If leukocytes were the predominant cell type, the sample was determined to be in the diestrus stage. If nucleated cells were abundant, the sample was considered to be in the proestrus stage. If cornified squamous epithelial cells were the predominant cell type, the sample was determined to be in the estrus stage. If squamous epithelial cells and leukocytes were abundant, the sample was considered to be in the metestrus stage. Mice already in estrus were sacrificed to eliminate the effect of the estrous cycle on the rest of the experiment.

### Hormone assays

The serum testosterone (T) concentrations of the mice were determined using commercial iodine [^125^I] radioimmunoassay kits (North Institute, Bio-Tech, Beijing, China). The intra- and inter-assay errors among all assays were <10% and 15%, respectively. The sensitivity limit of testosterone was 0.02 ng/mL.

### Histological staining

Ovaries were sectioned at 4 μm, with 40 μm discarded between every section, and six sections were collected from each ovary. Sections were stained with hematoxylin and eosin (H&E) according to standard histological procedures and analyzed by conventional light microscopy. The examination was performed by two histologists who were unaware of the source of the material. Follicles were classified according to Kauffman et al. (22).

### RNA sequencing analysis in the ovarian tissues of a DHEA plus HFD-induced PCOS mouse model

Ovarian tissues from PCOS and control mice were taken, and total RNA was extracted by the TRIzol method. RNA sequencing (RNA-seq) was conducted by Beijing Allwegene Technology Company Limited (Beijing, China). The cDNA library was constructed by polymerase chain reaction (PCR). RNA-seq was performed using the PE150 sequencing strategy of Illumina’s second-generation high-throughput sequencing platform. Poor quality RNA-seq reads and adapters were filtered out. Clean read data were aligned using Tophat2 and Cufflinks software to complete the transcriptome comparison (23).

### Acquisition and preparation of data from public databases

Gene expression profiles from PCOS patients were obtained from the Gene Expression Omnibus (GEO; https://www.ncbi.nlm.nih.gov/geo/). In this study, the eight datasets from PCOS patients were GSE1615, GSE5850, GSE10946, GSE34526, GSE98595, GSE102293, GSE137684 and GSE168404. Three datasets from rats are GSE108499, GSE83220 and GSEGS59456. Three datasets from mice are GSE156895, GSE103056 and GSE171431. These data are shown in **Table 1**. Using the exprSet function in the “limma” package (version 3.42.2), the data were normalized to produce the expression matrix. The sample consisted of the PCOS group and the control group. Genes associated with PCOS were obtained from the PCOSKB database (http://pcoskb.bicnirrh.res.in/), a compilation of molecular, biochemical and clinical databases on PCOS (24).

**Table 1:** Details of microarray and RNA-seq datasets.

Accession ID	Platform	Platform ID	Number of patients	Symbol (N/P)	Organism	Modelling method
GSE1615	Affymetrix HG-U133A	GPL96	9	4/5	Homo sapiens	NA
GSE5850	Affymetrix HG-U133A 2.0	GPL570	12	6/6	Homo sapiens	NA
GSE10946	Affymetrix HG-U133A 2.0	GPL570	23	11/12	Homo sapiens	NA
GSE34526	Affymetrix HG-U133A 2.0	GPL570	10	3/7	Homo sapiens	NA
GSE98595	Affymetrix HG-1_0-st	GPL6244	8	3/5	Homo sapiens	NA
GSE102293	Affymetrix HG-U133A 2.0	GPL570	6	4/2	Homo sapiens	NA
GSE137684	Agilent SurePrint G3 GE 8x60K	GPL17077	12	4/8	Homo sapiens	NA
GSE168404	Illumina HiSeq 2500	GPL16791	10	5/5	Homo sapiens	NA
GSE108499	RiboArray Rat mRNA	GPL24411	12	3/3	Rattus norvegicus	TBT
				3/3		BPA
				3/3		TBT plus BPA
GSE83220	Illumina HiSeq 2500	GPL18694	4	2/2	Rattus norvegicus	HFHS
GSE59456	Affymetrix Rat 230 2.0	GPL1355	8	4/4	Rattus norvegicus	DHT
GSE156895	Illumina HiSeq 2000	GPL13112	5	2/3	Mus musculus	PNA
GSE103056	Affymetrix Mouse 430 2.0	GPL1261	2	1/1	Mus musculus	PNA
GSE171431	Affymetrix MTA-1_0	GPL20258	6	3/3	Mus musculus	DHT
Our data	Illumina NovaSeq	–	6	3/3	Mus musculus	DHEA plus HFD

TBT, tributyltin; BPA, bisphenol A; DHT, dihydrotestosterone; PNA, prenatally androgenized; DHEA, dehydroepiandrosterone; HFD, high fat diet; HFHS, high fat high sugar; NA, not available.

### Homologation of human, rat and mouse species

The useMart function in the “biomaRt” R package was used to establish a relationship with the Ensembl BioMart web service, and the datasets were selected by setting the dataset parameter to hsapiens_gene_ensembl, mmusculus_gene_ensembl and rnorvegicus_gene_ensembl (25). The 14 datasets obtained from the GEO database were intersected with our data to obtain the shared genes.

### Batch effect removal and consolidation of datasets

The Empirical Bayes (EB) method was used to remove batch effects among different datasets (26). Principal component analysis (PCA) was performed to visualize the results of batch effect removal using EB. Datasets with the same animal model method were merged, while eight datasets of PCOS patients were also merged.

### DEGs and pathway enrichment analyzes

For each group of PCOS animal models and PCOS patients, DEGs were screened by comparing the PCOS group with the control group using Student’s *t* test. The DEGs were identified according to *P* < 0.05, *t* value > 0 for up-regulated genes and *t* value < 0 for down-regulated genes. To identify the functions of the DEGs, Gene Ontology (GO) enrichment analysis was performed for three categories: biological processes (BP), cellular component (CC) and molecular function (MF). Kyoto Encyclopedia of Genes and Genomes (KEGG) pathway enrichment analysis was performed to determine the function of DEGs.

### Protein–protein interaction network

Protein-protein interaction (PPI) network analysis of the DEGs was conducted using the STRING online biological database (https://string-db.org/) with a threshold value of ≥ 0.4 (medium confidence). PPI networks were constructed and visualized using Cytoscape software (http://cytoscape.org/). The Molecular Complex Detection (MCODE) and CytoHubba plug-ins were utilized to analyze the modules and hub genes of the PPI network in Cytoscape, respectively. The GeneCards database (https://www.genecards.org/) was searched to determine the functions of the hub genes.

### Statistical analysis

Student’s *t* test and false discovery rate (FDR) were applied in the identification of DEGs. The “sva” package (version 3.34.0) is used for batch effect removal. The “biomaRt” R package (version 2.42.1) was used for homology processing among different species. The “clusterProfiler” R package (version 3.14.3) was used to cluster the DEG enrichment pathways, and the “GOplot” package (version 1.0.2) and “ggplot2” (version 3.3.5) were used to visualize the results of the enrichment analysis. Statistical analysis was performed using R software (version 3.6.3). In the animal experiments, Student’s *t* test was used to analyze the serum testosterone concentration, and GraphPad Prism 9 was used for plotting. All data are presented as the mean ± SD. A two-sided *P* value < 0.05 indicated that the difference was statistically significant.

## Results

### DHEA plus HFD induced the formation of polycystic ovaries

H&E staining showed increased numbers of atretic and cystic follicles and decreased numbers of corpora lutea in the ovarian tissues of PCOS mice compared with control mice (*P* < 0.05, *P* < 0.001; **Supplementary Figures 1A–C**). Furthermore, an irregular estrous cycle (**Supplementary Figures 1E, F**) and elevated serum testosterone levels (*P* < 0.001; **Supplementary Figure 1D**) were observed in this model compared with the control, which indicated that the PCOS model was successfully constructed. RNA-seq analysis was performed on three ovarian tissue samples in each group of control and PCOS patients, and DEGs were screened out.

### Data preprocessing and batch effect removal

**Figure 2:**
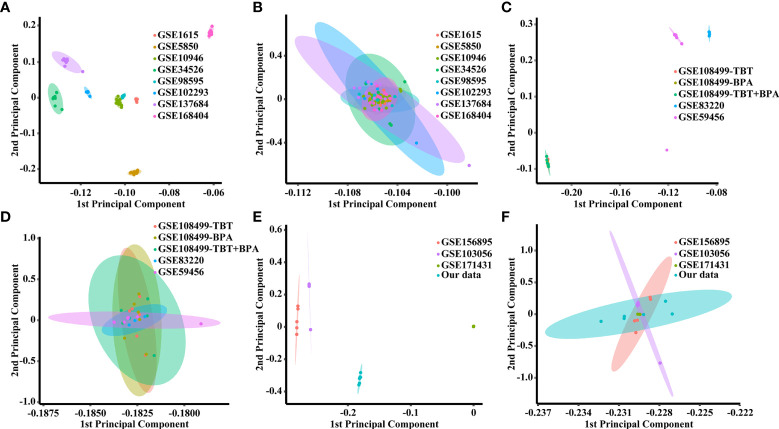
PCA score plots for classifying samples from different datasets. **(A)** Human datasets before batch effect removal. **(B)** Human datasets after batch effect removal by the EB method. **(C)** Rat datasets before batch effect removal. **(D)** Rat datasets after batch effect removal by the EB method. **(E)** Mouse datasets before batch effect removal. **(F)** Mouse datasets after batch effect removal by the EB method.

The homology analysis of the datasets is shown in **Supplementary Table 1**, and 5724 shared genes were obtained from the intersection. As shown in **Figures 2A, C, E**, there were batch effects among the different datasets. The EB method was used to remove batch effects and cluster all samples together, as shown in **Figures 2B, D, F**. The datasets generated using the same model approach were merged, and the eight datasets from PCOS patients were also merged. Then, data from eight groups of PCOS animal models and PCOS patients were obtained. The model approach, metabolic changes and reproductive abnormalities of the eight groups of PCOS animal models are shown in **Table 2**.

**Table 2:** Information about the eight groups PCOS animal models.

Method	Specie	Treatment time	Estrus cycle	CL	AF	Atr F	CF	FSH	LH	T	E2	Body weight	Ovary body index	Abnormal metabolism	Reference
**TBT**	SD rats	16d	irregular	↓	↓	↑	↑	NS	NA	NA	NA	NA	↓	lipid metabolism disorder	(27)
	Wistar dams	30d	irregular	NA	NA	↑	↑	NA	NA	↑	↓	↑	↓	abnormal lipid accumulation	(28)
**BPA**	SD rats	16d	irregular	↓	↓	↑	↑	NA	NA	NA	NA	NA	↓	lipid metabolism disorder	(27)
	SD rats	10d	irregular	↓	↓	↑	↑	NS	↑	↑	↑	NS	↓	NA	(29)
**TBT plus BPA**	SD rats	16d	irregular	↓	↓	↑	↑	NA	↑	↑	NA	NA	↓	lipid metabolism disorder	(27)
**HFHS**	SD rats	11w	irregular	↓	NA	↑	↑	NS	NS	NA	NA	↑	NS	insulin resistance	(18)
	SD rats	14w	irregular	↓	NA	NS	↑	NA	↓	↑	↓	↑	NS	insulin resistance altered steroidogenesis	(17)
**DHT**	C57BL/6J mice	70d	irregular	↓	↓	↑	↑	↓	↑	NS	↑	↑	NS	hypercholesterolemia	(30)
	SD rats	90d	irregular	↓	↓	↓	↑	↑	↑	↓	↓	↑	↓	insulin resistance	(31)
**PNA**	ICR mice	gestational days 16–18	irregular	↓	↑	↑	↑	NA	↑	↑	↑	↑	↑	abnormal folate one-carbon metabolism	(32)
	SD rats	gestational days 16–19	irregular	↓	↓	↑	↑	NA	↑	↑	↑	NS	NS	insulin resistance	(33)
**DHEA plus HFD**	SD rats	20d	irregular	↓	↓	NA	↑	NS	↑	↑	↑	↑	NS	lipid metabolism disorder impaired glucose tolerance	(16)
	C57BL/6J mice	20d	irregular	↓	NA	↑	↑	↑	↑	↑	NS	↑	NS	lipid metabolic disorders	(21)

SD, Sprague-Dawley; CL, corpora lutea; AF, antral follicles; Atr F, atretic follicles; CF, cystic follicles; FSH, follicle-stimulating hormone; LH, luteinizing hormone; E2, estradiol; T, testosterone; NA, not available; NS, no significance ; ↓, reduce;↑, increase.

### Intersection of DEGs in PCOS animal models and patients

**Figure 3:**
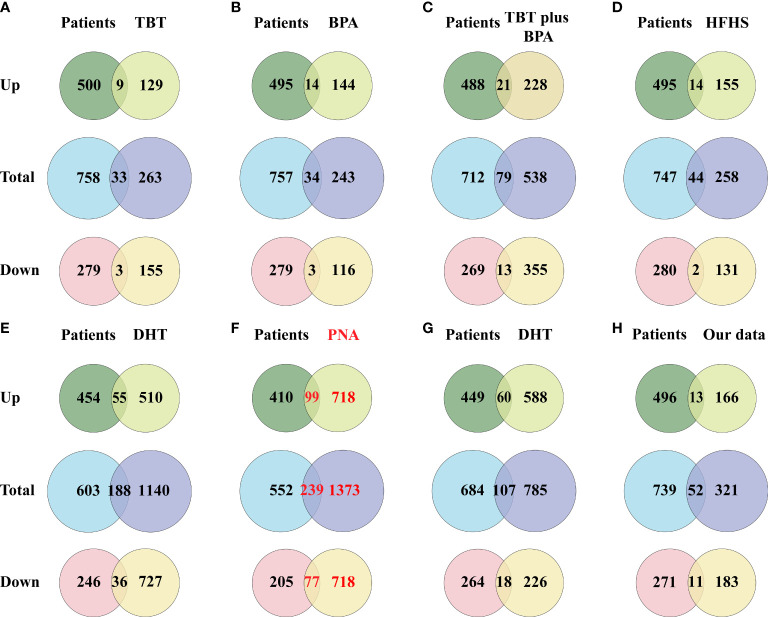
Venn diagram showing the intersection of DEGs (including up-regulated genes and down-regulated genes) between the PCOS animal modeland patients. **(A)** DEGs of the TBT-induced PCOS rat model. **(B)** DEGs of the BPA-induced PCOS rat model. **(C)** DEGs of the TBT plus BPAinduced PCOS rat model. **(D)** DEGs of the HFHS-induced PCOS rat model. **(E)** DEGs of the DHT-induced PCOS rat model. **(F)** DEGs of the PNA PCOS mouse model. **(G)** DEGs of the DHT-induced PCOS mouse model. **(H)** DEGs of the DHEA plus HFD-induced PCOS mouse model.

**Figure 4:**
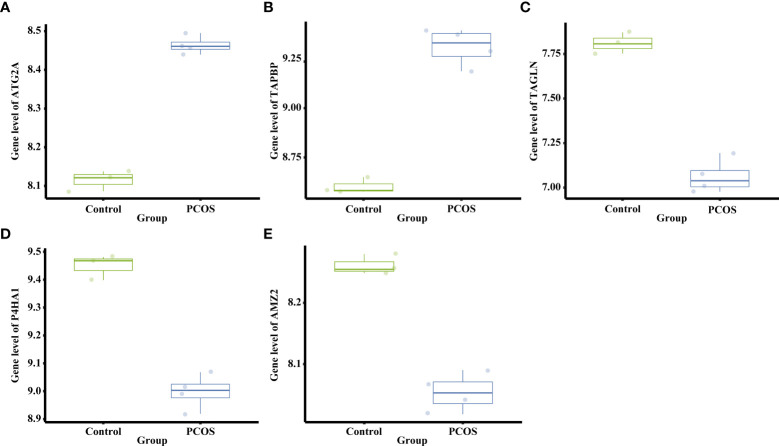
Differential mRNA expression of the top five DEGs shared between PNA mice and PCOS patients. **(A)** The gene level of *Atg2a*. **(B)** The gene level of *Tapbp*. **(C)** The gene level of *Tagln*. **(D)** The gene level of *P4ha1*. **(E)** The gene level of *Amz2*.

A total of 791 DEGs, including 509 up-regulated and 282 down-regulated genes, were identified in PCOS patients compared with controls (**Supplementary Table 2**). The DEGs from the eight groups of animal models of PCOS was compared with those of PCOS patients (**Figure 3**). The results showed 239 shared genes between prenatally androgenized (PNA) mice and PCOS patients, including 99 upregulated and 77 downregulated DEGs, as shown in **Figure 3F**. DHT-induced PCOS rats and mice shared fewer DEGs with PCOS patients than PNA mice. The 188 and 107 overlapping genes in DHT-induced PCOS rats and mice are shown in **Figures 3E, G** respectively. The top five DEGs shared by PNA mice and PCOS patients were *Atg2a*, *Tapbp*, *Tagln*, *P4ha1* and *Amz2*, as illustrated in box plots in **Figure 4**.

### Intersection between DEGs of PCOS animal models and genes from PCOSKB

**Figure 5:**
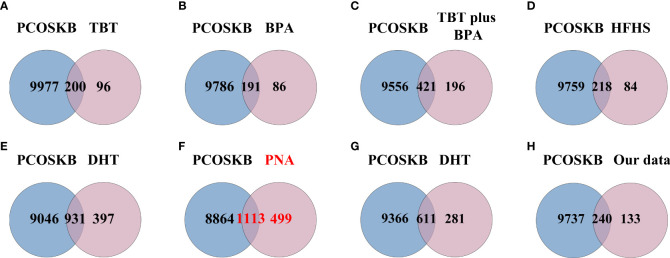
Venn diagram showing the intersection of PCOS animal models and genes from PCOSKB. **(A)** DEGs of the TBT-induced PCOS rat model. **(B)** DEGs of the BPA-induced PCOS rat model. **(C)** DEGs of the TBT plus BPA-induced PCOS rat model. **(D)** DEGs of the HFHS-induced PCOS rat model. **(E)** DEGs of the DHT-induced PCOS rat model. **(F)** DEGs of the PNA PCOS mouse model. **(G)** DEGs of the DHT-induced PCOS mouse model. **(H)** DEGs of the DHEA plus HFD-induced PCOS mouse model.

The above results were confirmed when comparing the PCOS-related genes obtained from the PCOSKB database with the DEGs in the eight groups of PCOS animal models **Figure 5**. From the PCOSKB website, 9,977 genes associated with PCOS were obtained. Of these PCOS-related genes, 1113 were identified in PNA mice (**Figure 5F**). The next highest number of overlapping genes were observed in DHT-induced PCOS rats and mice, with 931 and 611 DEGs shared with PCOS-associated genes, respectively (**Figures 5E–G**).

### Intersection of pathway enrichment analyzes in PCOS animal models and patients

**Figure 6:**
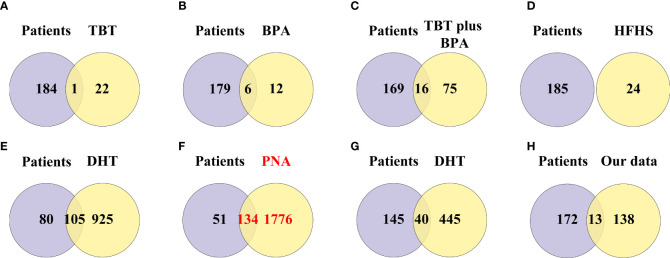
Venn diagram showing the intersection of pathway enrichment analyzes between PCOS animal models and patients. **(A)** GO and KEGG terms enriched in the TBT-induced PCOS rat model. **(B)** GO and KEGG terms enriched in the BPA-induced PCOS rat model. **(C)** GO and KEGG terms enriched in the TBT plus BPA-induced PCOS rat model. **(D)** GO and KEGG terms enriched in the HFHS-induced PCOS rat model. **(E)** GO and KEGG terms enriched in the DHT-induced PCOS rat model. **(F)** GO and KEGG terms enriched in the PNA PCOS mouse model. **(G)** GO and KEGG terms enriched in the DHT-induced PCOS mouse model. **(H)** GO and KEGG terms enriched in the DHEA plus HFD-induced PCOS mouse mod.

**Figure 7:**
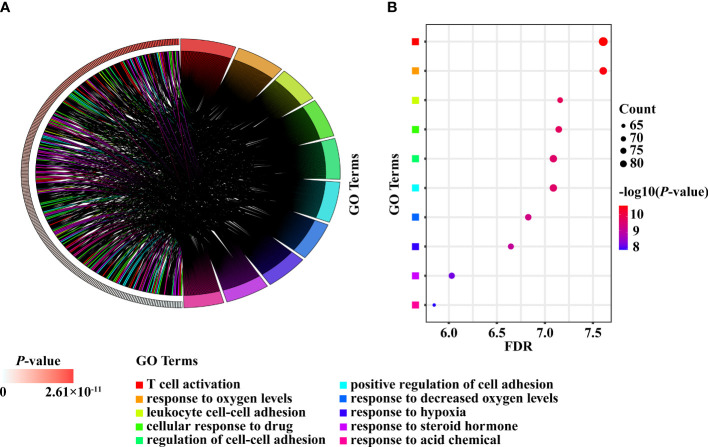
The top ten enriched pathways shared between PCOS patients and PNA mice. **(A)** Chord diagram. **(B)** Bubble diagram.

A total of 791 DEGs from PCOS patients were selected for GO and KEGG pathway enrichment analyzes, and 185 pathways were obtained. The comparison showed that 134 of the pathways enriched in PNA mice were the same as those enriched in PCOS patients (**Figure 6F** and **Supplementary Table 3**). The top ten pathways of PNA in ascending order of FDR value are shown in **Figure 7**. PCOS patients shared 105 pathways with DHT-induced rats and 40 pathways with DHT-induced mice. The top ten pathways selected from each model are shown in **Supplementary Figures 2**, **3**.

### PPI network analysis of DEGs

**Figure 8:**
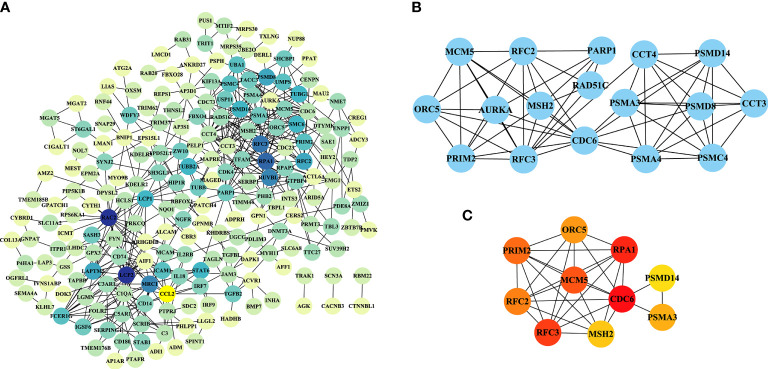
PPI network analysis of DEGs in PNA mice. **(A)** A PPI network with 211 nodes and 492 edges was constructed using Cytoscape software. **(B)** The top module results of MCODE PPI analysis. **(C)** The ten genes with the greatest linkage were selected for treatment as hub genes.

The PPI networks based on the intersection of DEGs between PCOS animal models and patients were downloaded from the STRING website and visualized using Cytoscape software. The PPI network for the PNA mice is shown in **Figure 8A**, which was composed of 211 nodes and 492 edges. The top module, with a maximum rating of 7.5, is shown in **Figure 8B**, created by the MCODE plug-in. The ten most highly connected genes in this PPI network were selected as hub genes (*Cdc6*, *Rpa1*, *Rfc6*, *Mcm5*, *Prim2*, *Rfc2*, *Orc5*, *Psma3*, *Msh2* and *Psmd14*) in **Figure 8C**. DHT-induced rats and mice were also individually submitted to PPI network analysis in **Supplementary Figures 4** and **5**, respectively.

## Discussion

Although there are many studies involving patients with PCOS, animal models have been used as the basis of pathophysiological research (8). More than 30 animal models of PCOS have been reported, but most of the models express only certain PCOS phenotypes or exhibit additional relevant features that are generally beyond the scope of the syndrome (9). In our study, the modelling approach, metabolic changes and reproductive abnormalities of the eight models used in PCOS research were taken into consideration (**Table 2**). Because the DHEA plus HFD-induced animal model is highly similar to PCOS in terms of metabolism and phenotypic characteristics (21), we used this method to establish a PCOS mouse model. We further employed RNA-seq analysis of ovarian tissue from PCOS patients and controls and included these results for analysis together with the seven datasets mentioned above.

We analyzed the consistency between eight PCOS animal models and patients at the mRNA level in terms of DEGs obtained from the GEO database, PCOS-related genes obtained from the PCOSKB website and pathways obtained by GO and KEGG pathway enrichment analyzes. This study confirmed that the PNA mouse model can best simulate patients in terms of DEG and pathway enrichment analyzes, followed by the DHT-induced PCOS rat and mouse models. The PNA model was prepared by subcutaneous or intramuscular injection of testosterone, testosterone propionic acid, DHT, and DHT propionic acid at different doses and different stages of pregnancy, from early-to-mid to late gestation (19), and the offspring rats were used as subjects in this study.

Based on the data of PNA mice in GSE103056 and GSE156895 from the relevant literature (34, 35), the subcutaneous injection of 70 μl of sesame oil alone or containing 350 μg of DHT per day on days 16-18 of gestation in females was used for model induction, and the offspring of the treated mice were considered the target PNA mice. This leads to the conclusion that, among the existing modelling methods, DHT treatment may be the optimal method for constructing rodent models of PCOS. Maternal treatment of study subjects may be a better model than direct DHT-induced PCOS in rats and mice. PCOS is known to have a tendency to run in families (36) and is a highly complicated genetic disorder (37). Hyperandrogenic gestation in the uterus can lead to the development of PCOS in adult offspring (38, 39), which may account for the superiority of PNA mice over DHT-induced rats and mice. This result also suggests that genetic factors influencing PCOS should be considered when selecting an animal model of PCOS, and it also provides clues for subsequent mechanistic studies.

The top ten DEGs of PNA mice, including *Axl, Atg2a, Klhl5, Mafb, Brix, Gpc3, Cdc42ep3, Prkag2, Porcn* and *Haus5*, were associated with autophagy, immunity, cytokinesis, cytoskeleton and inflammation. *Atg2a, TAPBP, Tagln, P4ha1, Amz2, Arhgdib, Cybrd1, Tmem185b, Prim2* and *Ets2* are the top ten DEGs with concordance in PCOS patients and PNA mice. These genes are associated with apoptosis, senescence, inflammation, autophagy and cell signaling. There is evidence that the rate of granular cell apoptosis is significantly increased in the antral follicles of women with PCOS compared to normal controls (40, 41). Apoptosis of follicular granulosa cells may therefore also be involved in the abnormal development of follicles associated with PCOS (42). PCOS is an inflammatory disease characterized by persistent nonspecific low-grade inflammation (43, 44). It has been shown that dietary induction stimulates the inflammatory response of monocytes in women with PCOS (45). There is a genetic basis for the inflammation observed in PCOS (46). Interestingly, *Atg2a* is shared by PNA mice and PCOS patients, but no studies of this gene relevant to PCOS are available. Meanwhile, *Klhl5, Mafb, Brix, Cdc42ep3, Haus5, TAPBP, Tagln, P4ha1, Arhgdib, Tmem185b, Prim2* and *Ets2* are all mentioned on the PCOSKB website. The GO and KEGG pathway enrichment analyses in the PNA mice showed that the top ten pathways in ascending order of FDR values were associated with chromosomes, lipid metabolism, membranes, and the mitotic cell cycle. A total of 134 GO and KEGG pathways enriched in PNA mice were shared with PCOS patients. The top ten pathways of PNA are shown in **Figure 7** and correlate with cell adhesion, T cells, hypoxia, steroid hormones and acidic chemicals. *Cdc6*, *Rpa1*, *Mcm5*, *Prim2*, *Orc5* and *Msh2* were associated with DNA replication in the PPI network analysis of PNA mice, which may also explain the greater likelihood of PCOS in the PNA mouse model. There is a chance that PNA mice may share not only reproductive but also immunity and hypoxia-related characteristics with PCOS patients.

*Alcam* and *Klf15* are in the top ten DEGs of both DHT-induced rat models and PCOS patients. The GeneCards website shows that *Alcam* and *Klf15* are associated with immunity and DNA replication, respectively. *Sgta* also possesses concordance, but studies have shown that *Sgta* is nominally significant (47) and has the potential to modulate androgen receptor signalling (48). In a DHT-induced PCOS rat model, GO and KEGG pathway enrichment analyzes showed that the top ten pathways were associated with metabolic processes, glandular development and intrauterine embryonic development through a variety of substances. As in PCOS patients, the top ten pathways were associated with hypoxia and metabolism of multiple substances. Glandular development and response to steroid hormones are present in both of the abovementioned groups.

*Mgst2, Cdkn1b, Tst, Mtmr3, Vdac1, Lpp, Pdhb, Pfkp, Hmga2* and *Bckdhb* are the top ten DEGs in the DHT-induced mouse model, and the GeneCards website shows that they are associated with inflammation, mitochondria and metabolism. One study suggests that *Lpp* may be a new candidate gene for PCOS (49). *Pfkp* was among the top ten DEGs of both the DHT-induced mouse model and PCOS patients. A low level of *Pfkp* expression in cumulus oocyte complexes was found in Chinese patients with PCOS, suggesting a potential link between cumulus oocyte complexes and reduced glycolysis in women with PCOS (50). Among them, *Mgst2, Cdkn1b, Lpp, Pdhb* and *Bckdhb* are all listed on the PCOSKB website. Pathway enrichment analysis in a DHT-induced mouse model revealed that the top ten results were associated with hypoxia, ketones, amino acids, amine metabolism, hematopoietic cell differentiation and regulation. Consistent with that in PCOS patients, the top ten pathways in DHT-induced mice showed correlation with mitochondria, hypoxia, vesicle lumen and amino acid metabolism.

Meanwhile, we observed that the DEGs and the enrichment pathways were different when lean and obese PCOS patients were compared with controls (51). The differentially enriched pathways of lean and obese PCOS patients included mitochondrial gene expression, cell adhesion and signal transduction, cell migration, ubiquitin catabolic process, inflammation and immune response. Our findings differ from those of *Idicula-Thomas* et al. Among them, mitochondria, cell adhesion, immunity and inflammation are consistent, but we also found that in the top ten pathways of the three better models, metabolism of various substances and hypoxia are also very important. This enriches the current understanding of PCOS. Although we did not consider the effect of obesity on PCOS in this study, we believe that this factor should be taken into account when constructing models.

In addition, several novel PCOS loci have been identified in genome-wide association studies (GWASs) in China and Europe (52–55). It has been shown that 14 independent loci, including ERBB4, THADA, and KRR1, are significantly associated with the risk of PCOS, and 11 of these loci may be associated with the endocrine and metabolic pathways in PCOS (56). We looked for novel PCOS loci in the DEGs of eight groups of animal models and found that the trend of *Irf1* expression was the same in the DHT rat model as in humans. The novel *Mapre1* locus showed the same trend in the PNA mouse model and in humans. Therefore, when studying PCOS candidate genes, it is necessary to compare the models with humans at the mRNA level to select an appropriate model.

However, some limitations exist in our study. First, it is important to mention that the GEO dataset for PCOS includes only rats and mice. There are no reports on other mammalian models, such as primates, which are more similar to humans. Second, the sample size of each model was relatively small; however, more data are expected to be uploaded to these public databases.

## Conclusion

In conclusion, the current study shows that among selected mouse and rat models, the PNA mouse model has the best consistency with PCOS patients at the mRNA level. Among the existing modelling methods, treatment with DHT may be the optimal method for constructing rodent models of PCOS. This study provides a new perspective for the evaluation of PCOS models and serves as a reference for researchers to select a more suitable animal model of PCOS.

## Data availability statement

The data analyzed in this study is subject to the following licenses/restrictions: The datasets used and analyzed during thecurrent study are available in the GEO (https://www.ncbi.nlm.nih.gov/geo/) repository. The dataset of DHEA plus HFD mouse model analyzed are not publicly available as it is still under study but are available from the corresponding author on reasonable request. Requests to access these datasets should be directed to Meijiao Wang, meijiaowang@cqmu.edu.cn.

## Ethics statement

The studies involving human participants were reviewed and approved by The Ethics Committees of Chongqing Medical University. Written informed consent for participation was not required for this study in accordance with the national legislation and the institutional requirements. The animal study was reviewed and approved by The Ethics Committees of Chongqing Medical University.

## Author contributions

JR and GT conceived the study. MW performed the animal experiments. JR analyzed the data and wrote the manuscript. WL and XR revised the figures and tables. MW, BX, JT, QP, and YW critically revised the manuscript. BX and MW made significant contributions to the conception and coordination. The authors read and approved the submitted version.

## Funding

The study was funded by National Natural Science Foundation of China (No. 82171624; 81972023), the Chongqing Natural Science Foundation (No. cstc2020jcyj-msxmX0294), Science and Technology Project of Chongqing Yuzhong District (No. 20200103), Scientific Research & Innovation Experiment Project of Chongqing Medical University (SRIEP202106; SRIEP202002) and the Chongqing Postdoctoral Science Foundation (No. cstc2021jcyj-bsh0029).

## Conflict of interest

The authors declare that the research was conducted in the absence of any commercial or financial relationships that could be construed as a potential conflict of interest.

## Publisher’s note

All claims expressed in this article are solely those of the authors and do not necessarily represent those of their affiliated organizations, or those of the publisher, the editors and the reviewers. Any product that may be evaluated in this article, or claim that may be made by its manufacturer, is not guaranteed or endorsed by the publisher.

## References

[1] Conway G Dewailly D Diamanti-Kandarakis E Escobar-Morreale HF Franks S Gambineri A. The polycystic ovary syndrome: a position statement from the European society of endocrinology. Eur J Endocrinol (2014) 171(4):P1–P29. doi: 10.1530/EJE-14-0253 24849517

[2] Fauser BCJM Chang J Azziz R Legro R Dewailly D Franks S. Revised 2003 consensus on diagnostic criteria and long-term health risks related to polycystic ovary syndrome (PCOS). Hum Reprod (2004) 19(1):41–7. doi: 10.1093/humrep/deh098 14688154

[3] Legro RS Kunselman AR Dunaif A. Prevalence and predictors of dyslipidemia in women with polycystic ovary syndrome. Am J Med (2001) 111(8):607–13. doi: 10.1016/S0002-9343(01)00948-2 11755503

[4] Escobar-Morreale HF. Polycystic ovary syndrome: definition, aetiology, diagnosis and treatment. Nat Rev Endocrinol (2018) 14(5):270–84. doi: 10.1038/nrendo.2018.24 29569621

[5] Bozdag G Mumusoglu S Zengin D Karabulut E Yildiz BO. The prevalence and phenotypic features of polycystic ovary syndrome: a systematic review and meta-analysis. Hum Reprod (2016) 31(12):2841–55. doi: 10.1093/humrep/dew218 27664216

[6] Ben-Shlomo I Younis JS. Basic research in PCOS: are we reaching new frontiers? Reprod Biomed Online (2014) 28(6):669–83. doi: 10.1016/j.rbmo.2014.02.011 24768413

[7] Hanna CW Demond H Kelsey G. Epigenetic regulation in development: is the mouse a good model for the human? Hum Reprod Update (2018) 24(5):556–76. doi: 10.1093/humupd/dmy021 PMC6093373 29992283

[8] Ryu Y Kim SW Kim YY Ku SY. Animal models for human polycystic ovary syndrome (PCOS) focused on the use of indirect hormonal perturbations: A review of the literature. Int J Mol Sci (2019) 20(11):27. doi: 10.3390/ijms20112720 PMC6600358 31163591

[9] Azziz R. PCOS: Animal models for PCOS - not the real thing. Nat Rev Endocrinol (2017) 13(7):382–4. doi: 10.1038/nrendo.2017.57 28474686

[10] Benrick A Chanclón B Micallef P Wu Y Hadi L Shelton JM. Adiponectin protects against development of metabolic disturbances in a PCOS mouse model. Proc Natl Acad Sci USA (2017) 114(34):E7187–96. doi: 10.1073/pnas.1708854114 PMC5576831 28790184

[11] Li Y Zheng Q Sun D Cui X Chen S Bulbul A. Dehydroepiandrosterone stimulates inflammation and impairs ovarian functions of polycystic ovary syndrome. J Cell Physiol (2019) 234(5):7435–47. doi: 10.1002/jcp.27501 30580448

[12] Jung JH Park HT Kim T Jeong MJ Lim SC Nah SY. Therapeutic effect of korean red ginseng extract on infertility caused by polycystic ovaries. J Ginseng Res (2011) 35(2):250–5. doi: 10.5142/jgr.2011.35.2.250 PMC3659527 23717068

[13] Raja MA Maldonado M Chen J Zhong Y Gu J. Development and evaluation of curcumin encapsulated self-assembled nanoparticles as potential remedial treatment for PCOS in a female rat model. Int J Nanomed (2021) 16:6231–47. doi: 10.2147/IJN.S302161 PMC8439717 34531655

[14] He Y Wang Q Li X Wang G Zhao J Zhang H. Correction: Lactic acid bacteria alleviate polycystic ovarian syndrome by regulating sex hormone related gut microbiota. Food Funct (2021) 12(10):4720–1. doi: 10.1039/D1FO90033A 33881104

[15] Esparza LA Schafer D Ho BS Thackray VG Kauffman AS. Hyperactive LH pulses and elevated kisspeptin and NKB gene expression in the arcuate nucleus of a PCOS mouse model. Endocrinology (2020) 161(4):bqaa018. doi: 10.1210/endocr/bqaa018 32031594 PMC7341557

[16] Zhang H Yi M Zhang Y Jin H Zhang W Yang J. High-fat diets exaggerate endocrine and metabolic phenotypes in a rat model of DHEA-induced PCOS. Reproduction (2016) 151(4):431–41. doi: 10.1530/REP-15-0542 26814210

[17] Volk KM Pogrebna VV Roberts JA Zachry JE Blythe SN Toporikova N. High-fat, high-sugar diet disrupts the preovulatory hormone surge and induces cystic ovaries in cycling female rats. J Endocr Soc (2017) 1(12):1488–505. doi: 10.1210/js.2017-00305 PMC5740526 29308444

[18] Roberts JS Perets RA Sarfert KS Bowman JJ Ozark PA Whitworth GB. High-fat high-sugar diet induces polycystic ovary syndrome in a rodent model. Biol Reprod (2017) 96(3):551–62. doi: 10.1095/biolreprod.116.142786 28203719

[19] Stener-Victorin E Padmanabhan V Walters KA Campbell RE Benrick A Giacobini P. Animal models to understand the etiology and pathophysiology of polycystic ovary syndrome. Endocrine Rev (2020) 41(4):39. doi: 10.1210/endrev/bnaa010 PMC7279705 32310267

[20] Walters KA Bertoldo MJ Handelsman DJ. Evidence from animal models on the pathogenesis of PCOS. Best Pract Res Clin Endocrinol Metab (2018) 32(3):271–81. doi: 10.1016/j.beem.2018.03.008 29779581

[21] Wang M Zhao D Xu L Guo W Nie L Lei Y. Role of PCSK9 in lipid metabolic disorders and ovarian dysfunction in polycystic ovary syndrome. Metabolism-Clinical Exp (2019) 94:47–58. doi: 10.1016/j.metabol.2019.02.002 30768966

[22] Kauffman AS Thackray VG Ryan GE Tolson KP Glidewell-Kenney CA Semaan SJ. A novel letrozole model recapitulates both the reproductive and metabolic phenotypes of polycystic ovary syndrome in female mice. Biol Reprod (2015) 93(3):69. doi: 10.1095/biolreprod.115.131631 26203175 PMC4710190

[23] Trapnell C Roberts A Goff L Pertea G Kim D Kelley DR. Differential gene and transcript expression analysis of RNA-seq experiments with TopHat and cufflinks. Nat Protoc (2012) 7(3):562–78. doi: 10.1038/nprot.2012.016 PMC3334321 22383036

[24] Joseph S Barai RS Bhujbalrao R Idicula-Thomas S. PCOSKB: A KnowledgeBase on genes, diseases, ontology terms and biochemical pathways associated with PolyCystic ovary syndrome. Nucleic Acids Res (2016) 44(D1):D1032–5. doi: 10.1093/nar/gkv1146 PMC4702829 26578565

[25] Durinck S Spellman PT Birney E Huber W. Mapping identifiers for the integration of genomic datasets with the R/Bioconductor package biomaRt. Nat Protoc (2009) 4(8):1184–91. doi: 10.1038/nprot.2009.97 PMC3159387 19617889

[26] Johnson WE Li C Rabinovic A. Adjusting batch effects in microarray expression data using empirical bayes methods. Biostatistics (2007) 8(1):118–27. doi: 10.1093/biostatistics/kxj037 16632515

[27] Yang Z Shi J Guo Z Chen M Wang C He C. A pilot study on polycystic ovarian syndrome caused by neonatal exposure to tributyltin and bisphenol a in rats. Chemosphere (2019) 231:151–60. doi: 10.1016/j.chemosphere.2019.05.129 31129395

[28] de Araujo JFP Podratz PL Sena GC Merlo E Freitas-Lima LC Ayub JGM. The obesogen tributyltin induces abnormal ovarian adipogenesis in adult female rats. Toxicol Lett (2018) 295:99–114. doi: 10.1016/j.toxlet.2018.06.1068 29908848

[29] Fernandez M Bourguignon N Lux-Lantos V Libertun C. Neonatal exposure to bisphenol a and reproductive and endocrine alterations resembling the polycystic ovarian syndrome in adult rats. Environ Health Perspect (2010) 118(9):1217–22. doi: 10.1289/ehp.0901257 PMC2944080 20413367

[30] Caldwell AS Middleton LJ Jimenez M Desai R McMahon AC Allan CM. Characterization of reproductive, metabolic, and endocrine features of polycystic ovary syndrome in female hyperandrogenic mouse models. Endocrinology (2014) 155(8):3146–59. doi: 10.1210/en.2014-1196 24877633

[31] Chen M-J Chou CH Chen SU Yang WS Yang YS Ho HN. The effect of androgens on ovarian follicle maturation: Dihydrotestosterone suppress FSH-stimulated granulosa cell proliferation by upregulating PPAR gamma-dependent PTEN expression. Sci Rep (2015) 5:18319. doi: 10.1038/srep18319 26674985 PMC4682139

[32] Lei L Ding L Su J Liu M Shi Q Zhou J. Attenuated expression of MTR in both prenatally androgenized mice and women with the hyperandrogenic phenotype of PCOS. PloS One (2017) 12(12):e0187427. doi: 10.1371/journal.pone.0187427 29232372 PMC5726624

[33] Wu XY Li ZL Wu CY Liu YM Lin H Wang SH. Endocrine traits of polycystic ovary syndrome in prenatally androgenized female sprague-dawley rats. Endocrine J (2010) 57(3):201–9. doi: 10.1507/endocrj.K09E-205 20057162

[34] Lei L Ding L Su J Liu M Shi Q Zhou J. Attenuated expression of MTR in both prenatally androgenized mice and women with the hyperandrogenic phenotype of PCOS. PloS One (2017) 12(12):15. doi: 10.1371/journal.pone.0187427 PMC5726624 29232372

[35] Qin YL Li T Zhao H Mao Z Ding C Kang Y. Integrated transcriptomic and epigenetic study of PCOS: Impact of Map3k1 and Map1lc3a promoter methylation on autophagy. Front Genet (2021) 12:14. doi: 10.3389/fgene.2021.620241 PMC7982605 33763111

[36] Dapas M Lin FTJ Nadkarni GN Sisk R Legro RS Urbanek M. Distinct subtypes of polycystic ovary syndrome with novel genetic associations: An unsupervised, phenotypic clustering analysis. PloS Med (2020) 17(6):28. doi: 10.1371/journal.pmed.1003132 PMC7310679 32574161

[37] Moghetti P Tosi F Bonin C Sarra DD Fiers T Kaufman JM. Divergences in insulin resistance between the different phenotypes of the polycystic ovary syndrome. J Clin Endocrinol Metab (2013) 98(4):E628–37. doi: 10.1210/jc.2012-3908 23476073

[38] Filippou P Homburg R. Is foetal hyperexposure to androgens a cause of PCOS? Hum Reprod Update (2017) 23(4):421–32. doi: 10.1093/humupd/dmx013 28531286

[39] Abbott DH Dumesic DA. Passing on PCOS: new insights into its epigenetic transmission. Cell Metab (2021) 33(3):463–6. doi: 10.1016/j.cmet.2021.02.008 PMC9133658 33657389

[40] Kumariya S Ubba V Jha RK Gayen JR. Autophagy in ovary and polycystic ovary syndrome: role, dispute and future perspective. Autophagy (2021) 17(10):2706–33. doi: 10.1080/15548627.2021.1938914 PMC8526011 34161185

[41] Paixao L Ramos RB Lavarda A Morsh DM Spritzer PM. Animal models of hyperandrogenism and ovarian morphology changes as features of polycystic ovary syndrome: a systematic review. Reprod Biol Endocrinol (2017) 15(1):12. doi: 10.1186/s12958-017-0231-z 28183310 PMC5301391

[42] Peng S-L Wu QF Xie Q Tan J Shu KY. PATL2 regulated the apoptosis of ovarian granulosa cells in patients with PCOS. Gynecolog Endocrinol (2021) 37(7):629–34. doi: 10.1080/09513590.2021.1928066 34008465

[43] Patel S. Polycystic ovary syndrome (PCOS), an inflammatory, systemic, lifestyle endocrinopathy. J Steroid Biochem Mol Biol (2018) 182:27–36. doi: 10.1016/j.jsbmb.2018.04.008 29678491

[44] Yang Y Xia J Yang Z Wu G Yang J. The abnormal level of HSP70 is related to Treg/Th17 imbalance in PCOS patients. J Ovarian Res (2021) 14(1). doi: 10.1186/s13048-021-00867-0 PMC8591891 34781996

[45] Gonzalez F Rote NS Minium J Kirwan JP. *In vitro* evidence that hyperglycemia stimulates tumor necrosis factor-a release in obese women with polycystic ovary syndrome. J Endocrinol (2006) 188(3):521–9. doi: 10.1677/joe.1.06579 16522732

[46] Escobar-Morreale HF Botella-Carretero JI Alvarez-Blasco F Sancho J San Millan JL. The polycystic ovary syndrome associated with morbid obesity may resolve after weight loss induced by bariatric surgery. J Clin Endocrinol Metab (2005) 90(12):6364–9. doi: 10.1210/jc.2005-1490 16189250

[47] Ewens KG Stewart DR Ankener W Urbanek M McAllister JM Chen C. Family-based analysis of candidate genes for polycystic ovary syndrome. J Clin Endocrinol Metab (2010) 95(5):2306–15. doi: 10.1210/jc.2009-2703 PMC2869537 20200332

[48] Butler MS Yang X Ricciardelli C Liang X Norman RJ Tilley WD. Small glutamine-rich tetratricopeptide repeat-containing protein alpha is present in human ovaries but may not be differentially expressed in relation to polycystic ovary syndrome. Fertility Sterility (2013) 99(7):2076–83.e1. doi: 10.1016/j.fertnstert.2013.01.140 23433514

[49] Zhang B Zhao H Li T Gao X Gao Q Tang R. Association study of gene LPP in women with polycystic ovary syndrome. PloS One (2012) 7(10):e46370. doi: 10.1371/journal.pone.0046370 23056290 PMC3463595

[50] Zhai Y,W Yao G Rao F Wang Y Song X Sun F. Excessive nerve growth factor impairs bidirectional communication between the oocyte and cumulus cells resulting in reduced oocyte competence. Reprod Biol Endocrinol (2018) 16(1):28. doi: 10.1186/s12958-018-0349-7 29580253 PMC5869770

[51] Idicula-Thomas S Gawde U Bhaye S Pokar K Bader GD. Meta-analysis of gene expression profiles of lean and obese PCOS to identify differentially regulated pathways and risk of comorbidities. Comput Struct Biotechnol J (2020) 18:1735–45. doi: 10.1016/j.csbj.2020.06.023 PMC7352056 32695266

[52] Chen Z-J Zhao H He L Shi Y Qin Y Shi Y. Genome-wide association study identifies susceptibility loci for polycystic ovary syndrome on chromosome 2p16.3, 2p21 and 9q33.3. Nat Genet (2011) 43(1):55–U75. doi: 10.1038/ng.732 21151128

[53] Day FR Hinds DA Tung JY Stolk L Styrkarsdottir U Saxena R. Causal mechanisms and balancing selection inferred from genetic associations with polycystic ovary syndrome. Nat Commun (2015) 6. doi: 10.1038/ncomms9464 PMC4598835 26416764

[54] Hayes MG Urbanek M Ehrmann DA Armstrong LL Lee JY Sisk R. Genome-wide association of polycystic ovary syndrome implicates alterations in gonadotropin secretion in European ancestry populations. Nat Commun (2015) 6:7502. doi: 10.1038/ncomms10762 26284813 PMC4557132

[55] Shi Y Karaderi T Jones MR Meun C He C Drong A. Genome-wide association study identifies eight new risk loci for polycystic ovary syndrome. Nat Genet (2012) 44(9):1020–5. doi: 10.1038/ng.2384 22885925

[56] Day F Karaderi T Jones MR Meun C He C Drong A. Large-Scale genome-wide meta-analysis of polycystic ovary syndrome suggests shared genetic architecture for different diagnosis criteria. PloS Genet (2018) 14(12). 10.1371/journal.pgen.1007813 PMC6300389 30566500

