# Supplementary material for: The PNA mouse may be the best animal model of polycystic ovary syndrome
Source: Front Endocrinol (Lausanne). 2022 Aug 8;13:950105. doi: 10.3389/fendo.2022.950105 (PMC9393894; doi:10.3389/fendo.2022.950105)
Supplement: Supplementary file 1 [file DataSheet_1.docx]

Supplementary Material


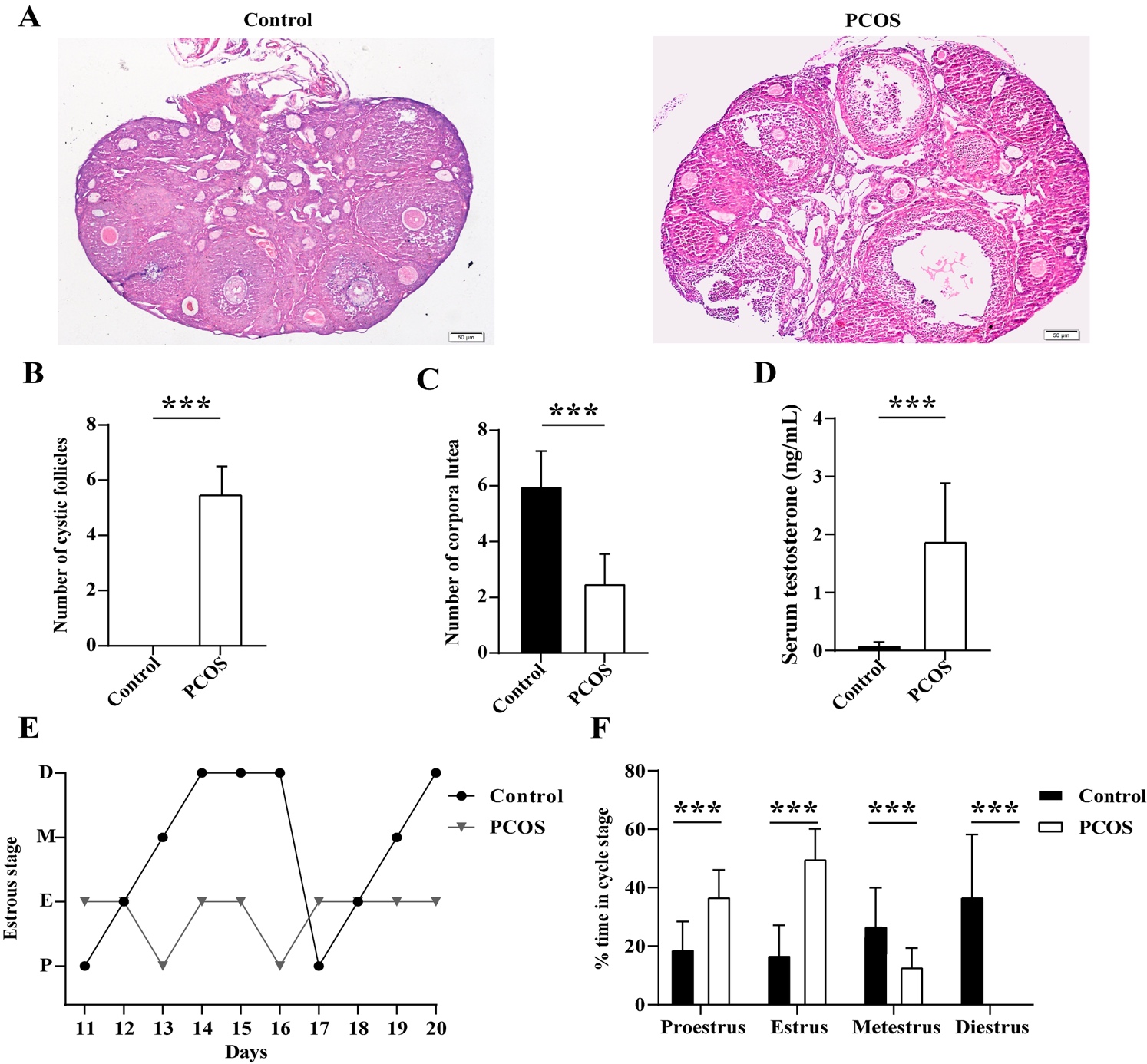


**Supplementary Figure 1.** The pathological changes in the DHEA plus HFD-induced PCOS mouse model. (A) Representative H&E staining of ovarian sections from each group, bar =50 mm. (B) Number of cystic follicles. (C) Number of corpora lutea. (D) Serum testosterone levels. Data are presented as the mean ± SD. ****P* < 0.001 denotes significant differences from the controls. (E) Representative estrous cycle of one mouse from each group. D, diestrus; M, metestrus; E, estrus; P, proestrus. (F) Percentage of female animals at each stage of the estrous cycle. Data are presented as the mean ± SD. ****P* < 0.001 denotes significant differences from the controls.

**
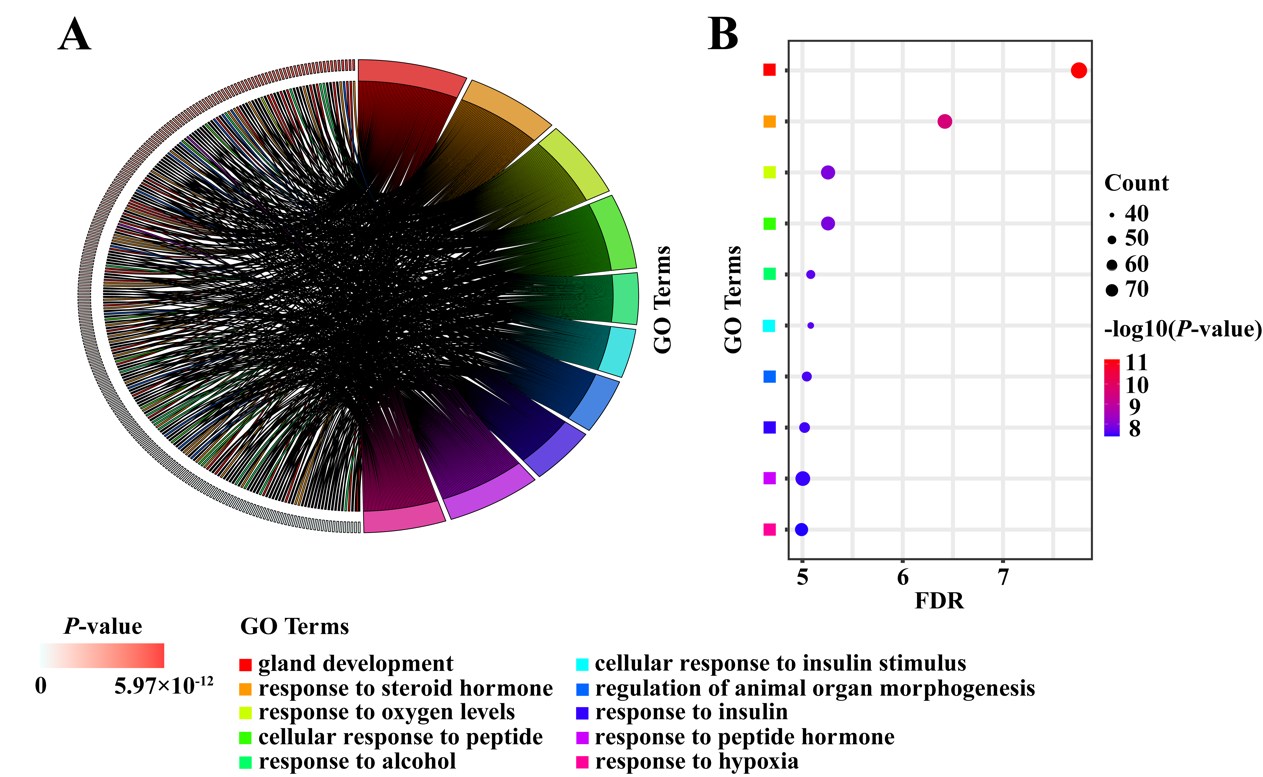
**

**Supplementary Figure 2.** The top ten enrichment pathways shared between PCOS patients and DHT-treated rats. (A) Chord diagram. (B) Bubble diagram.


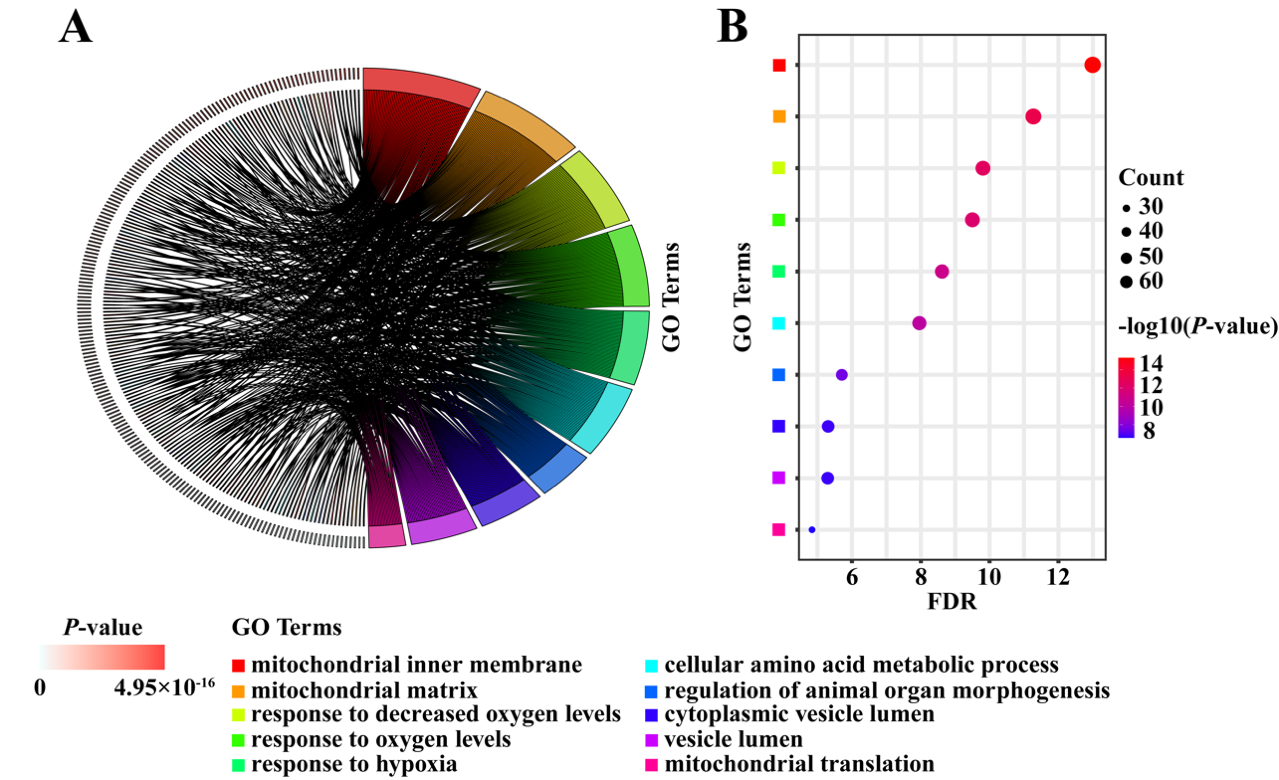


**Supplementary Figure 3.** The top ten enrichment pathways shared between PCOS patients and DHT-treated mice. (A) Chord diagram. (B) Bubble diagram.


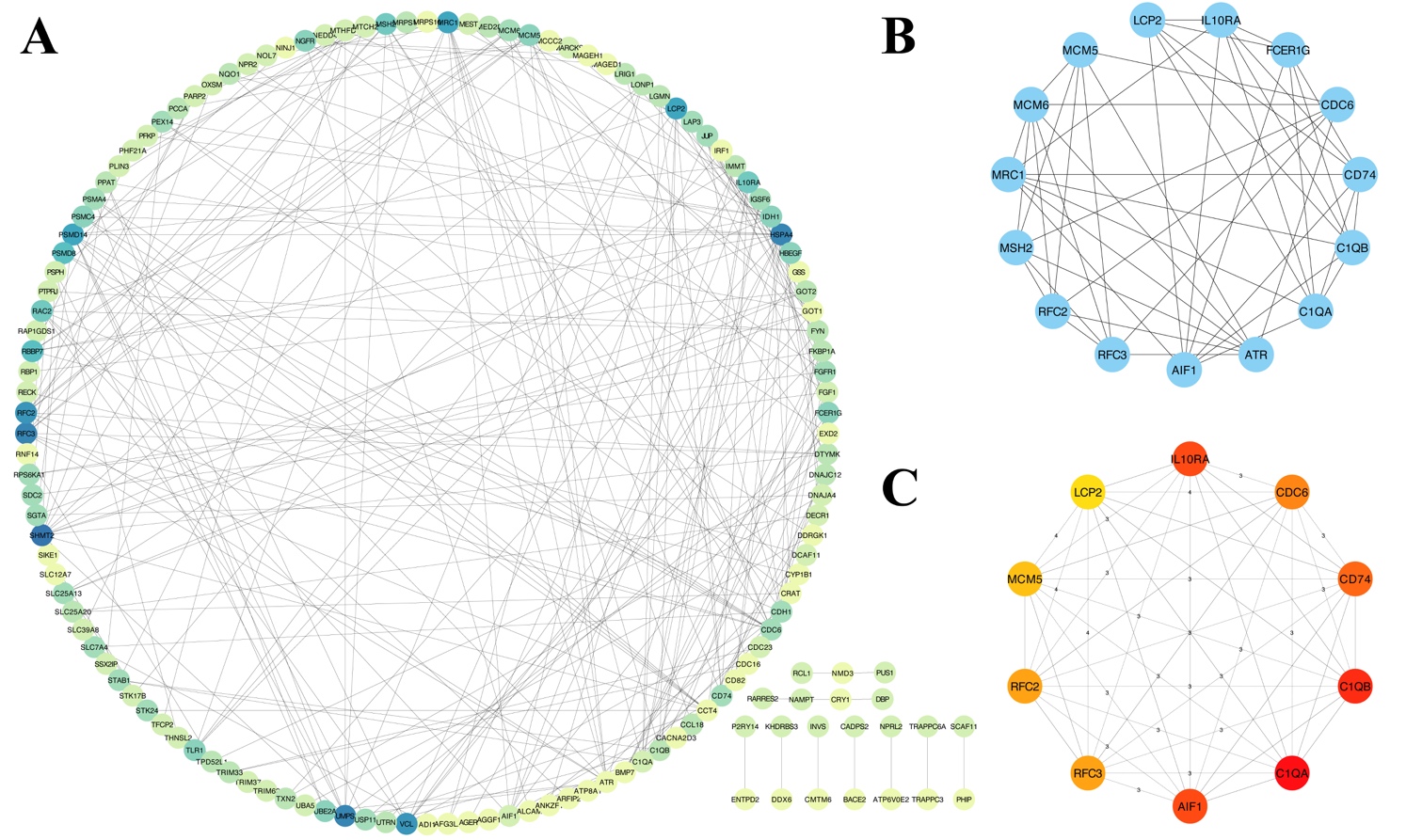


**Supplementary Figure 4.** PPI network analysis of DEGs in the DHT-treated rats. (A) A PPI network with 143 nodes and 282 edges was constructed using Cytoscape software. (B) The top module results of MCODE PPI analysis. (C) The ten genes with the greatest linkage were selected for treatment as hub genes.


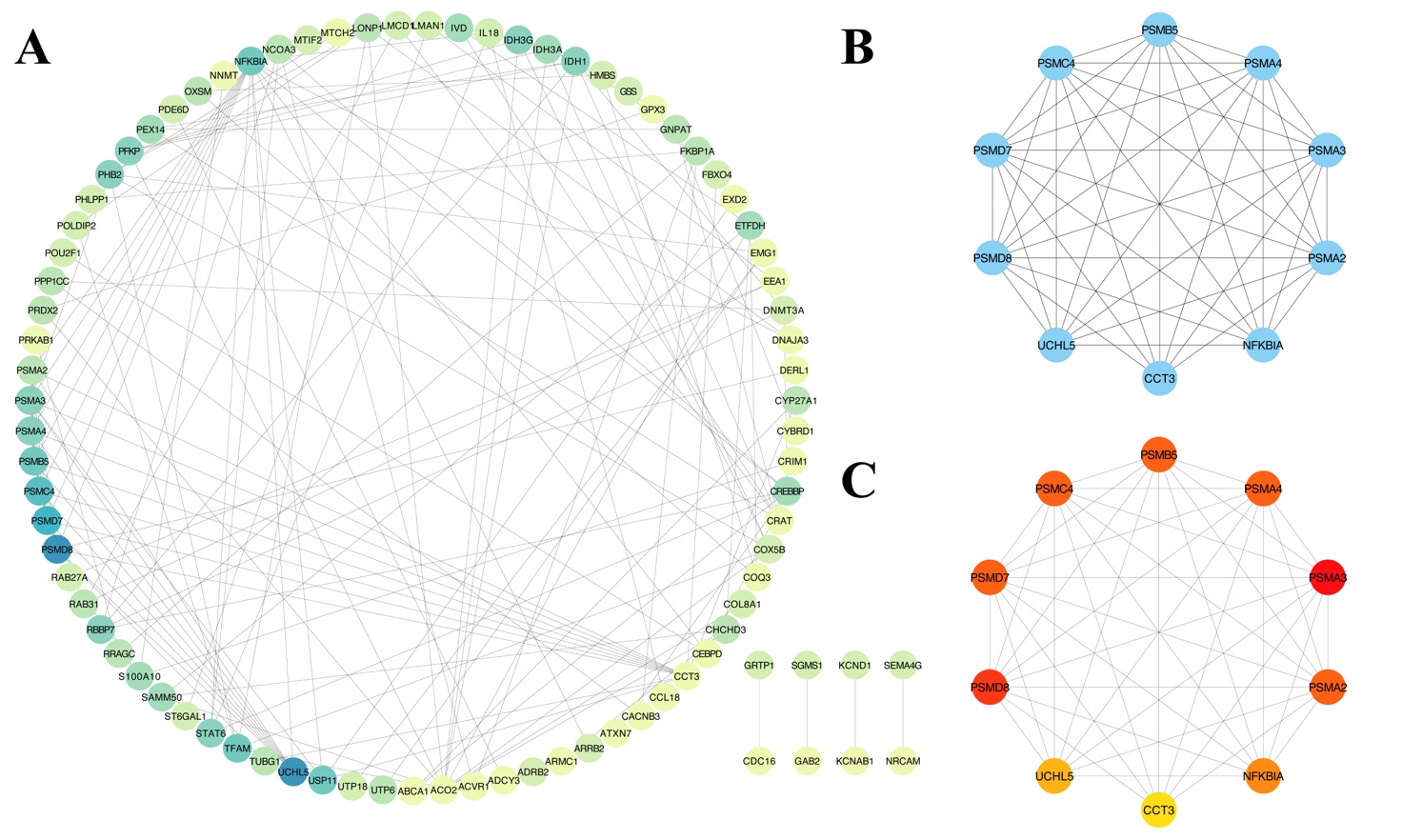


**Supplementary Figure 5.** PPI network analysis of DEGs in the DHT-treated PCOS mouse model. (A) A PPI network with 87 nodes and 155 edges was constructed using Cytoscape software. (B) The top module results of MCODE PPI analysis. (C) The ten genes with the greatest linkage were selected for treatment as hub genes.

**Supplementary Table 1.** Homolog identification in human, rat and mouse.

**Supplementary Table 2.** The statistical metrics for key DEGs of PCOS patients.

**Supplementary Table 3.** 134 of the pathways enriched in PNA mice were shared with PCOS patients.
