# Supplementary figures and images for: The PNA mouse may be the best animal model of polycystic ovary syndrome
Source: Front Endocrinol (Lausanne). 2022 Aug 8;13:950105. doi: 10.3389/fendo.2022.950105 (PMC9393894; doi:10.3389/fendo.2022.950105)

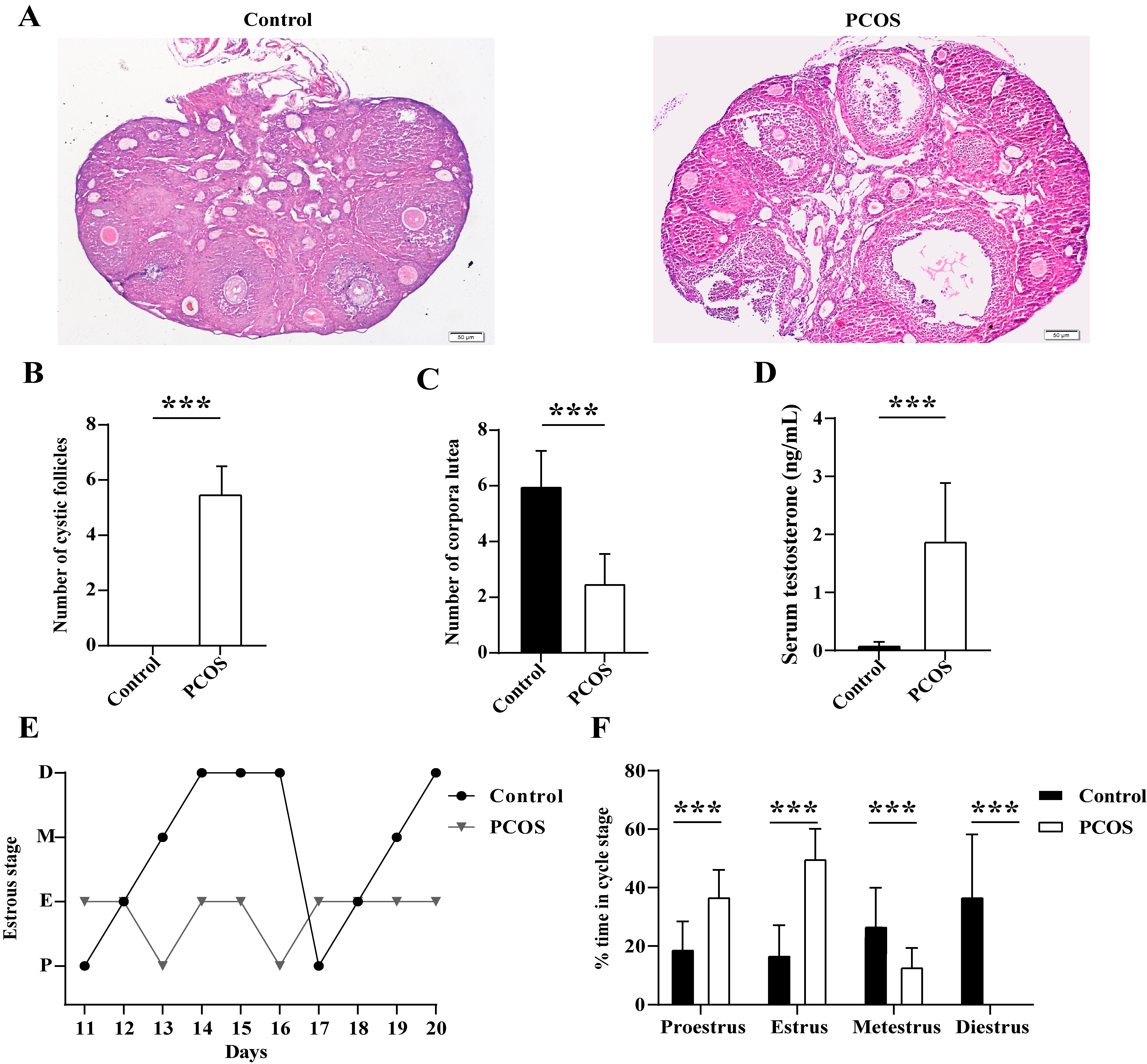

Supplement: Supplementary file 2 [file Image_1.tif]

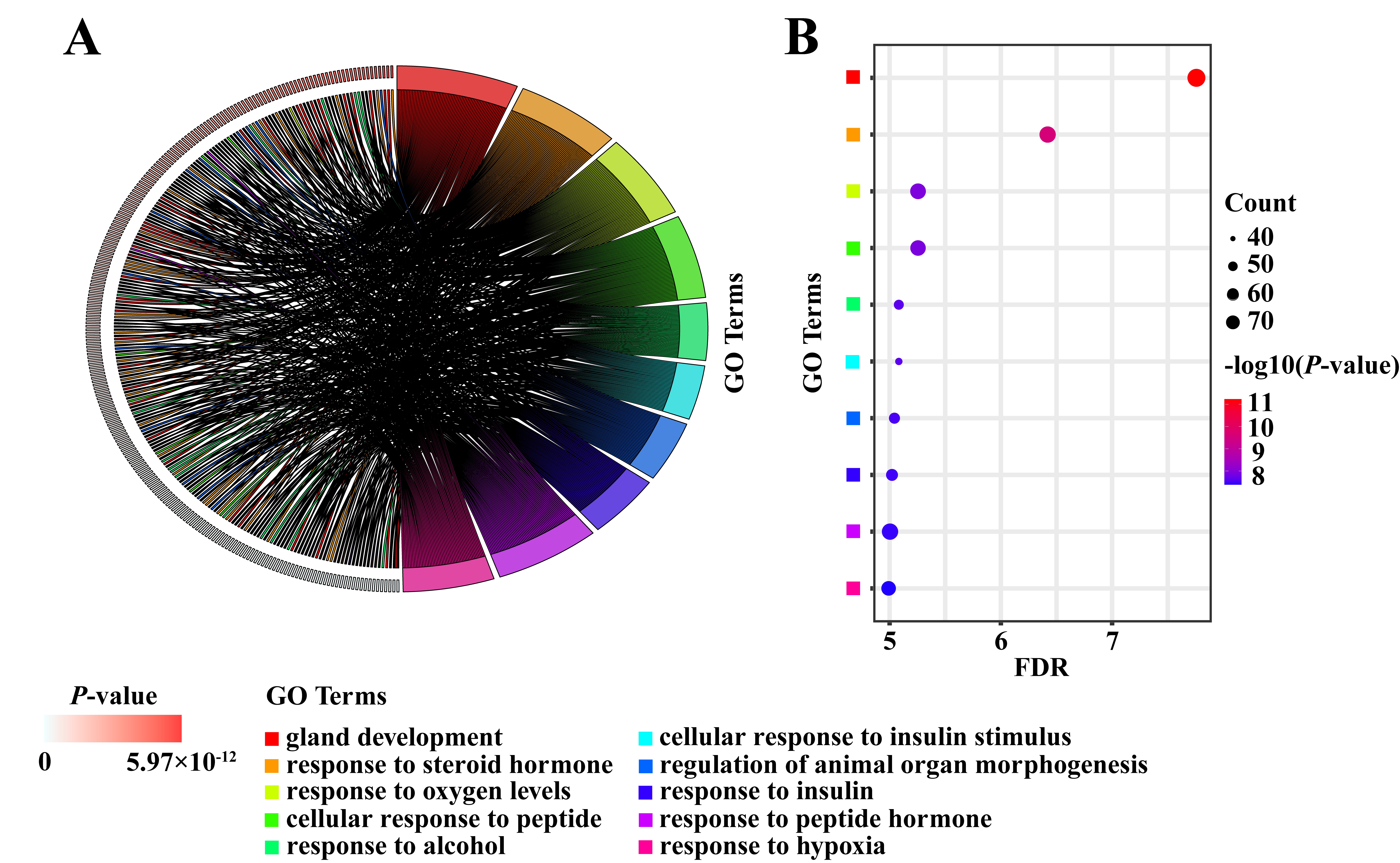

Supplement: Supplementary file 3 [file Image_2.tif]

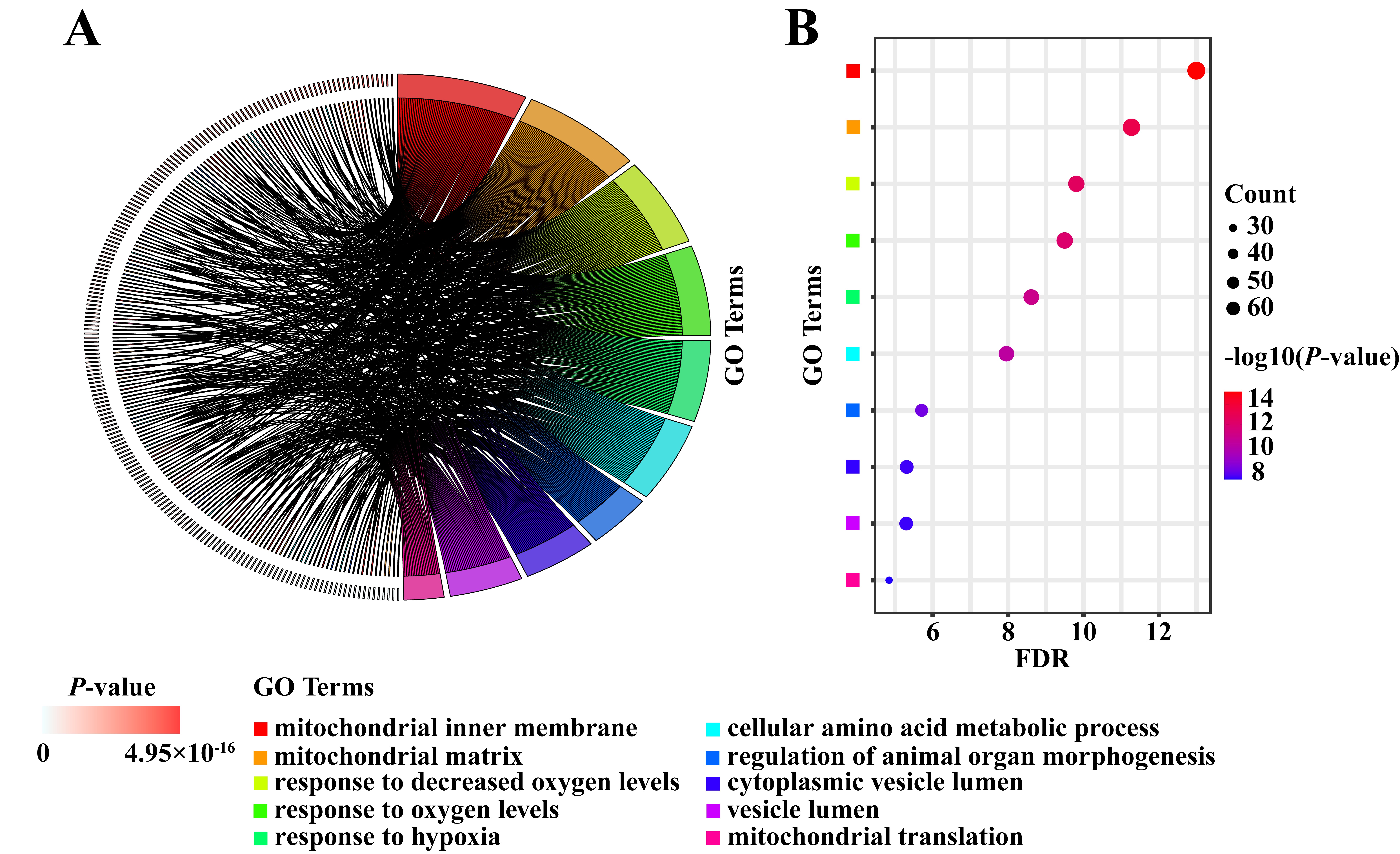

Supplement: Supplementary file 4 [file Image_3.tif]

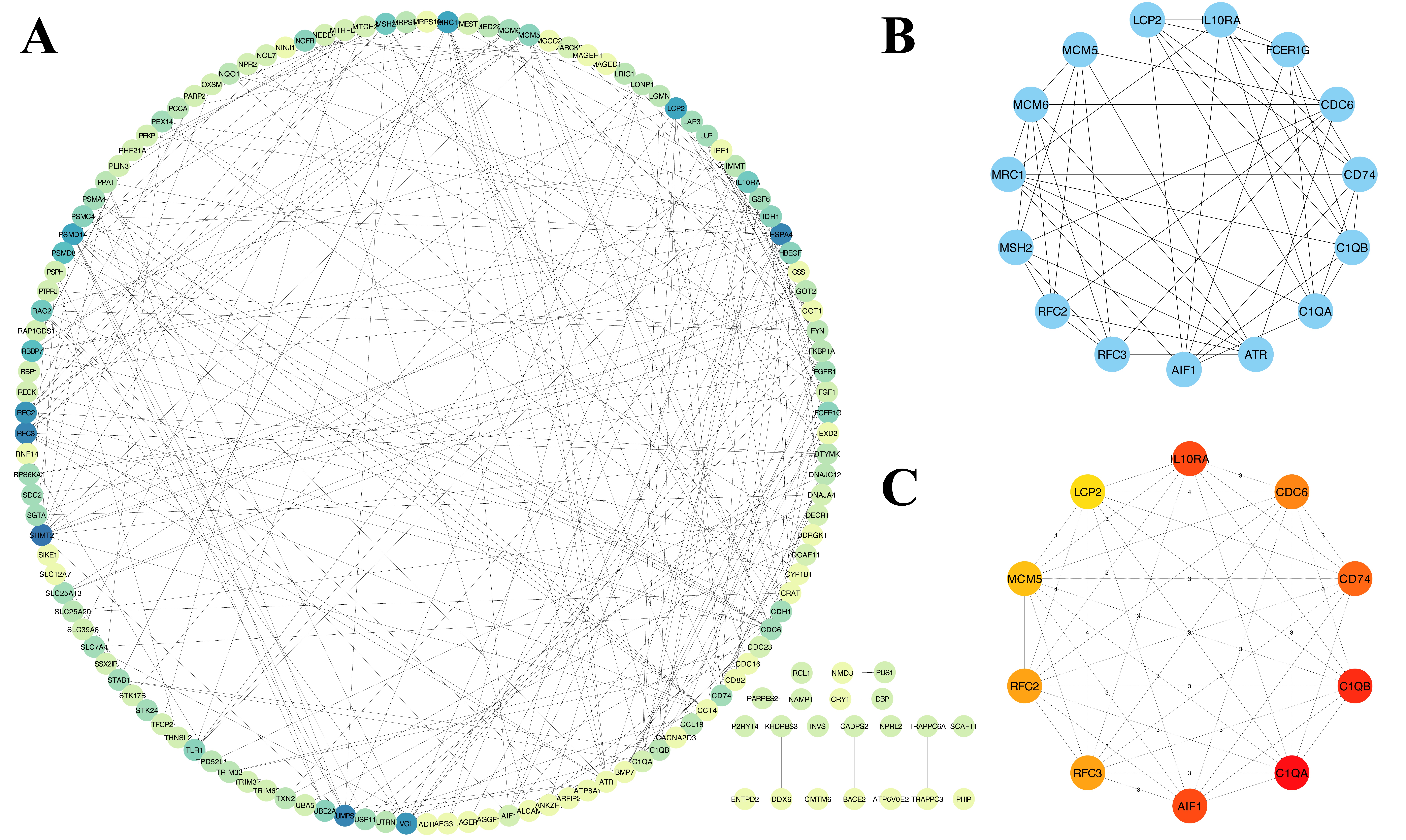

Supplement: Supplementary file 5 [file Image_4.tif]

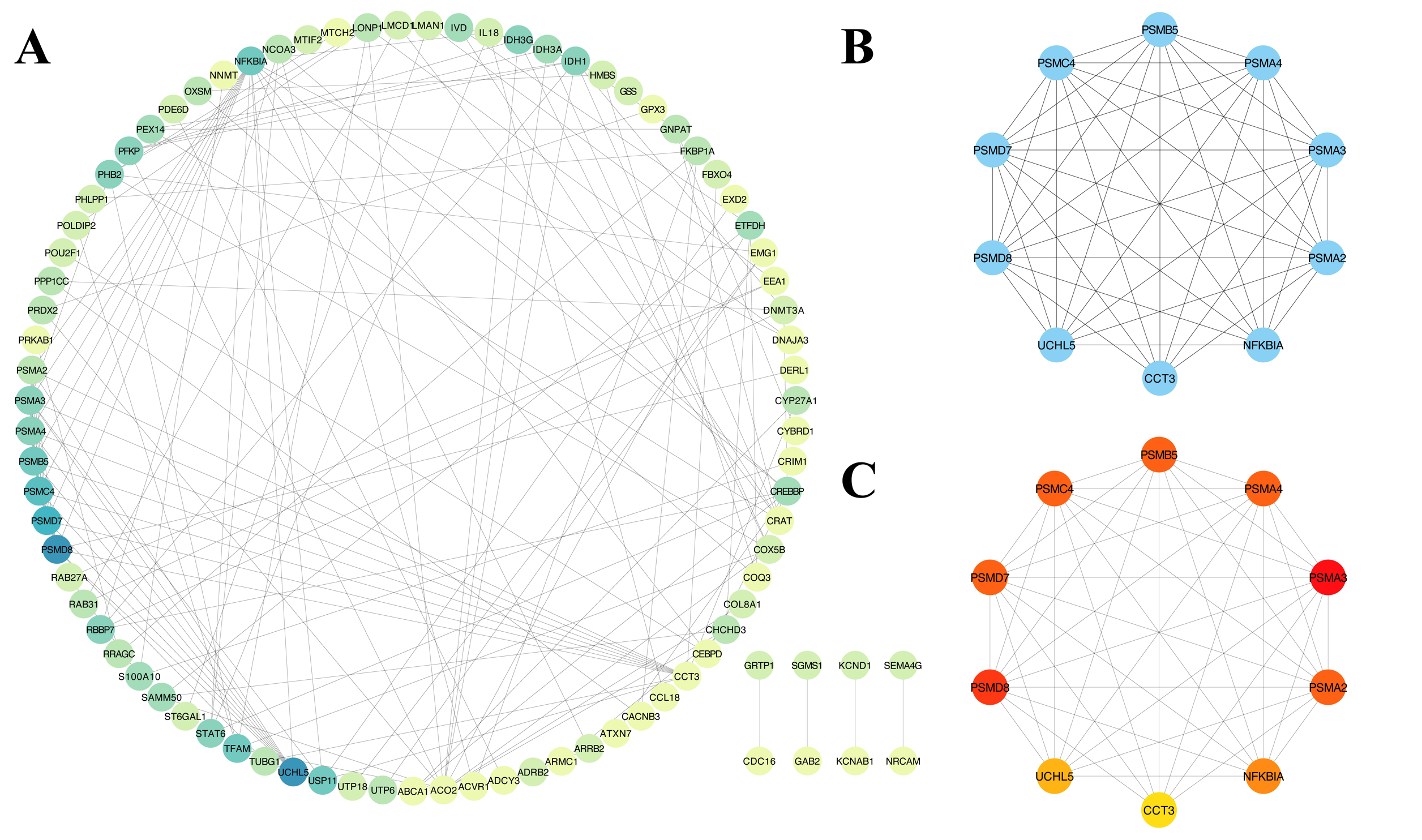

Supplement: Supplementary file 6 [file Image_5.tif]
